# COVID‐19 and cutaneous manifestations: A review of the published literature

**DOI:** 10.1111/jocd.15477

**Published:** 2022-11-07

**Authors:** Fabrizio Martora, Alessia Villani, Gabriella Fabbrocini, Teresa Battista

**Affiliations:** ^1^ Section of Dermatology – Department of Clinical Medicine and Surgery University of Naples Federico II Napoli Italy

**Keywords:** COVID‐19, dermatology, lesion or rash, skin manifestation, urticaria

## Abstract

**Background:**

COVID‐19 is a highly contagious respiratory tract infection caused by severe acute respiratory syndrome coronavirus 2. COVID‐19 outbreak, which caused thousands of deaths, has been declared a pandemic by the World Health Organization in March 2020.

**Aim:**

Skin manifestations related to SARS‐CoV‐2 infection can be divided mainly into five groups: chilblainlike lesions (CBLLs), maculopapular eruptions, urticarial eruptions, vesicular eruptions, and livedo or necrosis. Other skin findings reported are erythema multiforme (EM)‐like lesions and skin findings associated with multisystem inflammatory syndrome in children (MIS‐C) and rarely with multisystem inflammatory syndrome in adults (MIS‐A). Other manifestations such as pityriasis rosea or shingles are also reported.

**Methods:**

A total of 60 articles including reviews, studies and case reports were selected for the evaluation in this review.

**Results:**

The skin manifestations associated with COVID‐19 infection are numerous and can vary widely. The major dermatological patterns of COVID‐19 can be classified as inflammatory reactions (maculopapular/morbilliform, urticarial and vesicular rashes), or lesions of vascular origin (chilblain like rashes, petechiae/purpura, and livedo acemose‐like pattern)

**Conclusion:**

We believe that the dermatologist could play an important role in the response to the SARS‐CoV‐2 pandemic through early recognition of skin lesions suggestive of COVID‐19, particularly in paucisymptomatic infections where this recognition could direct toward an early diagnosis of infection that certainly leads to a better prognosis.

## 1 INTRODUCTION

On March 11, 2020, the World Health Organization (WHO) declared the new coronavirus disease (COVID‐19) a global pandemic.^1^ The disease caused by severe acute respiratory syndrome coronavirus 2 (SARS‐CoV‐2) was first identified in Wuhan, China, in December 2019, during these 3 years now it has become a serious threat to global public health.^2^

As of May 20, 2022, more than 520 912 257 confirmed cases of COVID‐19 worldwide since the start of the pandemic cases have been reported, with more than 6 272 408 deaths. Disease presentation can range from no symptoms to acute respiratory distress syndrome (ARDS), multiple organ failure to death. Common symptoms include fever, dry cough, fatigue, sputum production, shortness of breath, loss of sense of smell and taste, and conjunctivitis. Severe disease, on the contrary, is characterized by dyspnea, blood oxygen desaturation, respiratory failure, and venous thromboembolism.^3^, ^4^ To date, the considerable number of recently published case reports and clinical series have described a variety of skin manifestations associated with the infection; however, several explanations have been proposed on the mechanisms that induce the rash in COVID‐19 patients, primarily whether the virus can infect through an open skin wound or whether the skin manifestations are related to immune responses or finally whether the skin manifestations are caused by a newly prescribed drug.^5^, ^6^ However, research on the pathogenesis of the skin manifestations of COVID‐19 has not yet been carried out and is cause for study to date.

There are online registries, by the American Academy of Dermatology (AAD) and International League of Dermatological Societies (ILDS), in which skin lesions during or following SARS‐CoV‐2 infection can be reported to help describe the spectrum of SARS‐CoV‐2‐related skin change.^7^

The estimated incidence of cutaneous manifestations secondary to COVID‐19 is between 4% and 20.4%.^8^

The dermatologic manifestations with COVID‐19 may be related to the binding of SARS‐CoV‐2 to angiotensin‐converting enzyme‐2 (ACE2) receptors present in cutaneous blood vessels, eccrine gland epithelial cells, and the basal layer of hair follicles.^9^

Skin manifestations related to SARS‐CoV‐2 infection can be divided mainly into five groups: chilblain‐like lesions (CBLLs), maculopapular eruptions, urticarial eruptions, vesicular eruptions, and livedo or necrosis.^10^ Other skin findings reported are erythema multiforme (EM)‐like lesions and skin findings associated with multisystem inflammatory syndrome in children (MIS‐C) and rarely with multisystem inflammatory syndrome in adults (MIS‐A). Other manifestations such as pityriasis rosea or shingles are also reported.^11^, ^12^, ^13^

The aim of this review is to summarize the main patterns of dermatological manifestations associated with SARS‐CoV‐2 virus infection from the beginning of the pandemic to the present.There has often been confusion in the literature on infection‐associated manifestations, so for these reasons we selected only those published papers where many patients were involved and where an underlying pathogenetic mechanism was related that had obvious scientific relevance.

## 2 MATERIALS AND METHODS

We carried out a review of the English‐language literature up to June 20, 2022, related to the cutaneous manifestation of SARS‐CoV‐2 virus infection.

A search of the PubMed, Embase, and Cochrane Skin databases and that of clinicaltrials.gov was performed between the articles published in literature. The search terms were “Covid‐19,” “Skin manifestation,” “Dermatology,” “Urticaria,” “Erythema,” “‘lesions or rash,” “exanthema” (varicella‐like, papulo‐vesicular and morbilliform rash), and “vascular “(chilblain‐like, purpuric/petechial, and livedoid lesions). Only English‐language publications were selected. Then, a revision of the abstracts and texts of the articles was made independently by each author. As a result, a total of 63 articles including reviews, studies, and case reports were selected for the evaluation in this review.^14^

We excluded single case reports or articles where we did not consider the association with infection to be valid because the pathogenetic mechanism was not conclusive from a scientific point of view.

## 3 RESULTS

The skin manifestations associated with COVID‐19 infection are numerous and can vary widely. The major dermatological patterns of COVID‐19 can be classified as inflammatory reactions (maculopapular/morbilliform, urticarial and vesicular rashes), or lesions of vascular origin (chilblain like rashes, petechiae/purpura, and livedo acemose‐like pattern).^15^, ^16^ Almost 10% of the patients develop skin manifestations before the onset of respiratory illness, with cutaneous signs that can appear from few days before to several days after the diagnosis of COVID‐19.^10^ Skin manifestations can appear to both paucisymptomatic/asymptomatic patients and severe patients.^17^

In this section, we summarize the clinical, histological, and etiopathological features of the skin manifestations of COVID‐19.

### 3.1 Maculopapular lesions

Maculopapular lesions are the most prevalent cutaneous manifestations seen throughout the COVID‐19 pandemic, being observed up to 70% of patients.^18^ These lesions often follow viral infections or occur as adverse reactions to drugs.^19^ A case series of 375 patients with skin lesions associated with COVID‐19 identified a 47% prevalence of maculopapular lesions.^10^ Many of the maculopapular rashes reported were observed in middle‐aged patients.^20^, ^21^, ^22^ Anatomically, the majority of these lesions were located on the trunk of the body.^20^, ^21^, ^23^, ^24^, ^25^ The case series from Spain reported simultaneous onset of maculopapular lesions with COVID‐19′s symptoms,^10^ while other studies had noticed a later onset in their populations (average latency times of 27 days).^20^ The exanthems lasted for a short period (from 8.6 to 11.6 days).^10^, ^20^, ^21^ Galvan Casas et al. reported that pruritus was present in 56% of patients with maculopapular lesions and suggested that maculopapular rashes are associated with greater severity of COVID‐19 infections.^10^ Histopathology of these lesions differs depending on the time of onset.^22^ Early‐onset rashes show with epidermal spongiosis, a perivascular lymphocytic infiltrate and eosinophils in the dermis.^22^ While the histology of late‐onset lesions is characterized by mild superficial perivascular lymphocytic infiltrate and histiocytes among collagen fibers. These late‐onset lesions are devoid of mucin deposits.^22^, ^23^

A hypothesis on the etiopathology of these manifestations involves adverse drug reaction, considering that many drugs (e.g., chloroquine, hydroxicloroquine, lopinavir/ritonavir), used in the treatment of COVID‐19 infection are responsible for maculopapular rash^26^ and this agrees with the fact that lesions occur more frequently in patients with more severe infections and who have therefore taken more medication.^10^

Despite this, maculopapular eruptions also occurred in patients who had not taken any medications suggesting that these lesions may not solely be drug related.^21^, ^27^ Moreover, Herrero‐Moyano et al.^20^ hypothesized that these rashes could be caused by a cytokine storm produced by a hyperactive immune system against the virus.

### 3.2 Urticarial lesions

Urticarial lesions have also been noted in several COVID‐19 case series. They usually present as hives or angioedema and can be characterized as an erythematous rash followed by intense pruritic sensations.^28^ It is one of COVID‐19′s most frequent cutaneous manifestations. These lesions are typically distributed on the trunk or limbs.^10^, ^29^ In some cases, the rash can be generalized across the entire body or localized to the face.^23^ Onset is thought to occur at the same time as other systemic symptoms of COVID‐19 with an average duration of 6.8 days and more severe COVID‐19 cases.^10^ Pruritus is reported in 92% of patients with urticarial.^10^ Histopathological examination for urticarial lesions shows the presence of perivascular infiltrate of lymphocytes, with few eosinophils and upper dermal edema.^30^

Since many cases of urticaria have an association with therapy, drug‐induced exanthema could be a possible etiology of urticarial.^10^ Anti‐COVID‐19 drugs such as chloroquine, hydroxychloroquine, lopinavir/ ritonavir, corticosteroids, baricitinib, IVIG treatments, and checkpoint inhibitors have urticaria as a side effect.^26^ Moreover, another possible pathophysiological mechanism leading to urticaria could be the “cytokine storm.”^31^ Yet another possibility is the direct effect of the virus on the skin, considering the new data are about the direct skin localization of the virus.^32^

### 3.3 Chilblain‐like lesions

Chilblain‐like lesions (rarely bullous) represent late manifestations of COVID‐19 and, in contrast to other COVID‐19‐related skin manifestations, they are more often reported in children and young adults.

Chilblain lesions, also referred to as pernio, are a localized inflammatory skin disorder, thought to be induced by exposure to cold temperatures resulting in swelling, erythema, and violaceous coloration of the extremities such as fingers and/or toes.^33^ Chilblain‐like lesions or pernio‐like lesions represent late manifestations of COVID‐19 and, in contrast to other COVID‐19‐related skin manifestations, they are more often reported in children and young adults.^34^, ^35^, ^36^, ^37^, ^38^

The increased incidence of pernio‐like rashes and the temporal association with viral infection, has led to the coining of the term “COVID toes.” The prevalence of these lesions varies between studies. Freeman et al. documented 505 patients with dermatologic manifestations associated with COVID‐19, including 318 (63%) with pernio‐like lesions. Other studies have reported prevalence between 14.3% and 72%.^10^, ^23^, ^34^, ^35^, ^36^

These manifestations typically occur later in the course of the infection and last longer (for about a week or two on average) than erythematous rashes and usually appear in asymptomatic patients or with mild COVID‐19 disease.^10^, ^34^ As for clinical manifestations, pain, pruritus, and burning are the symptoms frequently recorded in patients with pernio‐like lesions.^34^, ^35^ Histopathological examination commonly displays focal vacuolar degeneration of the basal layer along with perivascular lymphocytic cuffs in the dermal regions and microthrombi.^22^, ^39^

The exact mechanism of chilblain‐like lesions is not fully understood as its presentation is unrelated to cold exposure. Pathogenesis behind chilblain could involve host viral response, vasculitis, vessel thrombosis, or neoangiogensis.^40^

Recent studies have not been able to associate chilblain‐like lesions with positive COVID‐19 infections; therefore, despite this, these lesions should not be considered an accurate indicator for diagnosis of COVID‐19 since there are studies that report chilblain‐like lesions in patients with no evidence of SARS‐COV2 infection by PCR or serology.^41^, ^42^

### 3.4 Vesicular lesions

Vesicular lesions among COVID‐19 patients are less common than the cutaneous manifestations mentioned above. The percentages reported in different studies range from 3.77% to 15%. These manifestations are typically seen in middle‐aged patients.^10^, ^23^, ^43^, ^44^ The trunk of the body is commonly affected in the localized forms of this type of rash; however, a diffuse pattern is described with polymorphic lesions also involving the extremities.^10^, ^23^, ^24^, ^43^, ^44^ The time of onset of cutaneous manifestations relative to other COVID‐19 symptoms varied between the few studies reported. The median time from COVID‐19 symptoms to vesicle eruption was 14 days (range 4–30 days).^10^, ^45^

Duration of rash reported by various studies ranged between 8 and 10 days.^10^, ^44^, ^45^ Vesicular lesions result to be associated with intermediate severity of COVID‐19.^10^, ^43^ Histopathological examination reveals the presence of intraepidermal vesicles associated with acantholysis, dyskeratosis, and ballooned keratinocytes^43^ along with lymphocytic perivascular infiltrate, vascular leak, and edema.^39^, ^43^, ^46^

The pathophysiologic mechanisms involved with vesicular lesions could be based on an overactivation of the immune system causing a “cytokine storm” affecting the skin.^30^ Another possible event behind the formation of vesicles is the direct cytopathic effect of SARS‐CoV‐2 on endothelium dermal vessels.^31^ As opposed to maculopapular and urticarial rashes, vesicular lesions associated with COVID‐19 are considered unrelated to antiviral medications.^43^ Moreover, vesicular lesions have been described as “specific cutaneous manifestations” of COVID‐19.^44^

### 3.5 Petechiae/purpura lesions

Petechiae/purpura rashes are among the less commonly described cutaneous manifestations in association with COVID‐19. De Masson et al.^23^ performed a retrospective study which reported that petechial patterns were present in only 3% of patients. Localization of the lesions included trunk and extremities. Petechiae/purpuric lesions onset is reported to be after COVID‐19 symptoms.^22^, ^47^ Various studies reported that palpable purpuric lesions are more frequent in middle‐aged patients recovering from severe COVID‐19 infections.^24^, ^47^ Histopathology shows interstitial and perivascular neutrophilia along with prominent leukocytoclasia.^47^

Proposed pathogenesis for petechiae/purpura skin lesions involves a pauci‐inflammatory thrombogenic vasculopathy. Magro et al^47^ examined skin tissues from three patients with severe COVID‐19 characterized by respiratory failure and purpuric skin rash that displayed a pauci‐inflammatory thrombogenic vasculopathy, with deposition of C5b‐9 and C4d in both involved and normally appearing skin. In addition, there was co‐localization of COVID‐19 spike glycoproteins with complement components.

Adverse dermatological effects associated with COVID‐19 drugs can be an aetiologic hypothesis, considering petechiae usually occur in patients with severe forms of COVID‐19. Finally, a direct cutaneous manifestation from SARS‐CoV‐2 could be a possibility considering petechiae are the result of other viral infections such as parvovirus B19 and dengue virus.

### 3.6 Livedoid eruption lesions

Livedoid eruptions are one of the least common cutaneous manifestations associated to COVID‐19. In a study of 375 COVID‐19 patients with cutaneous manifestations, only 6% of them presented with livedo reticularis‐like pattern.^10^ These lesions are generally localized on the trunk, flexor surface of forearms, dorsal hand, and dorsal foot.^10^, ^40^, ^48^ These lesions occurred at the same time as other COVID‐19 symptoms and appeared in elderly patients with severe infections. Mean duration of this lesions was 9.4 days.

As for the pathogenesis, the relationship of hypercoagulability to COVID‐19 infections is a possible mechanism, confirmed by the presence of higher D‐dimer and fibrin degradation product levels in patients with severe COVID‐19 and livedoid lesions.^49^ The mortality rate among patients with livedoid lesions resulted to be the highest of all cutaneous manifestations at 10%.^10^

### 3.7 Other rare skin manifestations

Multisystem inflammatory syndrome with Kawasaki disease‐like features is reported in children tested positive for positive for antibodies against SARS‐CoV‐2.^50^ It affects children older than Kawasaki disease and it is characterized by diffuse polymorphic rash, including maculo‐papular, erythema multiforme like, or diffuse erythroderma associated with fever, lymphadenopathy, strawberry tongue, and gastrointestinal symptoms.^51^

COVID‐19 is also associated to telogen effluvium (TE). Olds et al.^52^ retrospectively reviewed medical records of 552 patients with a diagnosis of COVID‐19 infection. Ten patients were identified with TE attributed to COVID‐19 infection, 90% female. On average, the hair shedding began 50 days after the first symptoms.^52^

Moreover, COVID‐19 may play a role in various immune‐related dermatologic conditions.

Alopecia areata is thought to be a dermatologic manifestation of COVID‐19, with cases most often appearing 1–2 months following infection.^53^

Other rare skin manifestations observed in COVID‐19 patients include pytiriasis rosea,^54^, ^55^ sebopsoriasis, Covid arm,^56^ herpes zoster,^57^ lichen planus^58^ painful ulcers on the hard palate and tongue, all fingernails onychopathy, diffuse pruritic pustular eruption, SDRIFE (symmetrical drug‐related intertriginous and flexural exanthema)‐like erythematous rash, pruriginous and painful subcutaneous nodular lesions, eruptive angiomas, and a pseudoherpetic variant of Grover disease.^27^

## 4 CONCLUSION

The purpose of our review was to describe the various skin manifestations that can be associated during COVID‐19 infection,^59^ although their underlying pathogenetic mechanisms are still unclear and are still under investigation. The possible relationship between skin manifestations and COVID‐19 is an important aspect that deserves great attention and efforts to improve the understanding of these phenomena.^60^, ^61^

SARS‐CoV‐2 infection is associated with an array of cutaneous manifestations which the dermatologists have tried to classify into various morphological groups and patterns. But a standard uniform classification does not exist and the role of SARS CoV‐2‐ direct or indirect in these lesions is still unclear and remains an open problem.

We believe that the dermatologist could play an important role in the response to the SARS‐CoV‐2 pandemic through early recognition of skin lesions suggestive of COVID‐19, particularly in paucisymptomatic infections where this recognition could direct toward an early diagnosis of infection that certainly leads to a better prognosis. Skin manifestations may be a prominent feature of COVID‐19 infection, and these lesions may be poorly recognized because of the lack of routine dermatologic consultations during the pandemic. For this reason, we consider the collections of all skin manifestations useful so that we can help colleagues in recognizing them.

Two years after the onset of the pandemic, the pathogenetic mechanisms that correlate skin manifestations with COVID‐19 infection are still unknown,^62^, ^63^, ^64^ and yet the appearance of new manifestations during infection highlights how this topic needs to be explored with further studies.

The role of dermatologists has been crucial as they have tried to classify the reactions into precise patterns ensuring the recognition of these lesions that allowed for correct diagnosis and consequently for optimal management.

The many reports in the literature have also allowed us to be able to explore the topic further during these 2 years by using the skin to explain yet unknown pathogenetic mechanisms.

However, there are still many unanswered questions, and further research will be needed in the future to better understand the relationship between COVID‐19 and skin manifestations.

## AUTHOR CONTRIBUTIONS

**Fabrizio Martora** and **Alessia Villani** helped in conceptualization, validation, visualization, writing—original draft preparation, and writing—review and editing. **Gabriella Fabbrocini helped in** conceptualization, validation, visualization, writing—review and editing, and supervision. **Teresa Battista** helped in supervision, conceptualization, validation, visualization, writing—original draft preparation, and writing—review and editing. **All authors read and approved the final version of the manuscript.**

## ETHICS STATEMENT

Authors declare human ethics approval was not needed for this study.

## Data Availability

Data sharing is not applicable to this article as no new data were created or analyzed in this study.
